# Evaluating Alzheimer's Disease Progression by Modeling Crosstalk Network Disruption

**DOI:** 10.3389/fnins.2015.00523

**Published:** 2016-01-19

**Authors:** Haochen Liu, Chunxiang Wei, Hua He, Xiaoquan Liu

**Affiliations:** Center of Drug Metabolism and Pharmacokinetics, China Pharmaceutical UniversityNanjing, China

**Keywords:** Alzheimer's disease, disease progression, crosstalk network, diagnosis, multi-marker

## Abstract

Aβ, tau, and P-tau have been widely accepted as reliable markers for Alzheimer's disease (AD). The crosstalk between these markers forms a complex network. AD may induce the integral variation and disruption of the network. The aim of this study was to develop a novel mathematic model based on a simplified crosstalk network to evaluate the disease progression of AD. The integral variation of the network is measured by three integral disruption parameters. The robustness of network is evaluated by network disruption probability. Presented results show that network disruption probability has a good linear relationship with Mini Mental State Examination (MMSE). The proposed model combined with Support vector machine (SVM) achieves a relative high 10-fold cross-validated performance in classification of AD vs. normal and mild cognitive impairment (MCI) vs. normal (95% accuracy, 95% sensitivity, 95% specificity for AD vs. normal; 90% accuracy, 94% sensitivity, 83% specificity for MCI vs. normal). This research evaluates the progression of AD and facilitates AD early diagnosis.

## Introduction

Alzheimer's disease (AD), the most common form of dementia, is characterized by a decline in cognitive ability (Ewers et al., 2012; Farah, 2014). Current estimates suggest that 36 million people worldwide have AD and the number is expected to almost triple in the next few decades (Grammas and Martinez, 2014). Since the symptomatic drugs currently on the market for AD have limited efficacy and only provides symptomatic relief without long-term cure, an important area to understand the disease progression and identify the potential vital pathological biomarkers for the progression has recently received increasing attention (Zhou et al., 2013; Salem et al., 2014).

Neuropsychological tests such as Mini Mental State Examination (MMSE) and Clinical Dementia Rating (CDR) are widely used in the clinical evaluation of patients with suspected dementia (Powell et al., 2006). However, neuropsychological tests alone are inadequate to diagnose AD at the early stages (Zamrini et al., 2004). The biomarker-based assessment of AD has been proposed to enhance the clinical detection of AD in early prodromal stages of the disease (Dubois et al., 2007). The use of biomarkers in clinical diagnostics may help us to determine whether some mild cognitive impairment (MCI) symptoms are due to AD (Ewers et al., 2012).^†^ Several researches have suggested that cerebrospinal fluid (CSF) based biomarkers are high precision risk factors in the disease process (Blennow and Hampel, 2003; Brys et al., 2009).

**Figure 1:**
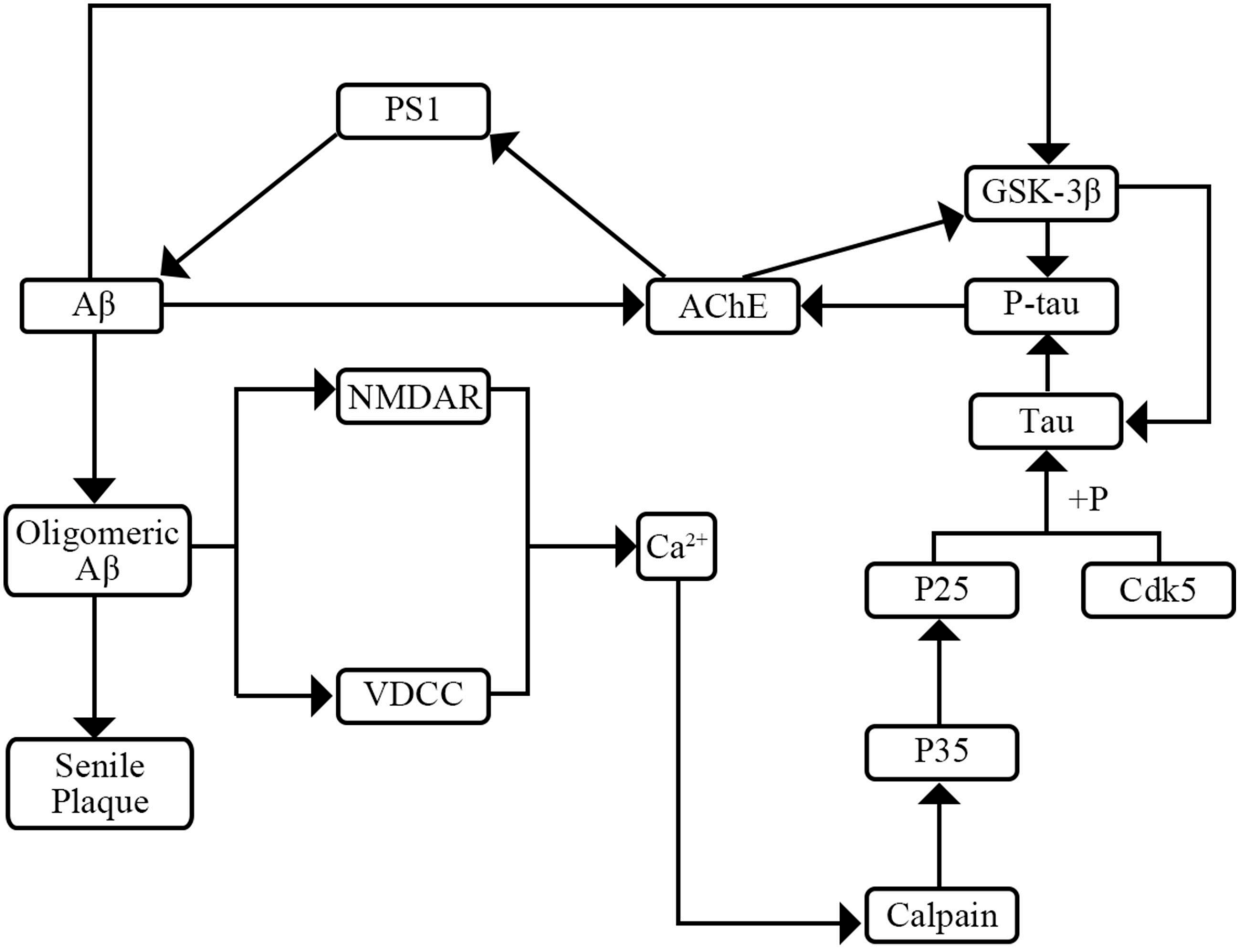
**The interaction of Aβ, tau, and P-tau**.

AD has two major pathological hallmarks in CSF including senile plaques and neurofibrillary tangles (NFT) (Kimura et al., 2014). NFTs make up from intracellular aggregates of hyperphosphorylated tau protein (P-tau) and senile plaques consist mainly of amyloid β peptide (Aβ) (Small, 2008; Kimura et al., 2014). Previous researches have shown the interaction of tau, P-tau, and Aβ (Figure 1). On one hand, P-tau can increase activity of acetyl-cholinesterase (AChE). Then the increased AChE activity can elevate Aβ production by modulating the levels of the γ-secretase catalytic subunit presenilin-1 (PS1) (García-Ayllón et al., 2011). On the other hand, Aβ may affect the level of P-tau through two pathways. Firstly, Aβ can raise the activity of AChE which can activate the tau kinase glycogen synthase kinase-3β (GSK-3β) inducing tau hyperphosphorylation (García-Ayllón et al., 2011). Secondly, Aβ can activate the voltage-dependent Ca^2+^ channels (VVCD) and N-methyl-D-aspartic acid (NMDA) receptors which results in the release of intracellular Ca^2+^ (Shen et al., 2006; Bezprozvanny and Mattson, 2008). Then the increased levels of intracellular Ca^2+^ might initiate a signal transduction pathway to activate Ca^2+^-sensitive protein kinases which are responsible for the hyperphosphorylation of tau (Shen et al., 2006). Furthermore, previous researches have expounded the role of Aβ and tau pathology. On one hand, Aβ plays a vital role in progression of AD which may lead in turn to a series of downstream events ranging from synapse loss to plaque deposition to inflammation to the triggering of tau hyperphosphorylation to the death of susceptible neurons (Herrup, 2010). On the other hand, tau pathology plays a complicated role in the progression of AD. Tau pathology may affect DNA repair, neuronal activity, and inter-neuronal signaling (Hanger et al., 2014). Though the mechanism and roles of tau pathology are not yet fully elucidated, a consensus that the tau pathology can enhance cognitive decline and cause dementia is widely accepted (Salminen et al., 2011).

In this study, a cross-talk network is established by integrating the interactions among CSF biomarkers. As a complex biology system, cross-talk network has two properties. The first is small-worldness which means that most pairs of nodes can be linked to each other by relatively short chains (Maslov and Ispolatov, 2007; Zhao et al., 2010). Secondly, robustness is the capacity of keeping homeostasis under a range of condition which may be disrupted by disease (He et al., 2013; Nijhout and Reed, 2014).

The aim of this research is to provide novel insight into the progression from the perspective of crosstalk network disruption. Nevertheless, cross-talk network is a large complicated system, evaluating the entire network robustness is less practical. To address this issue, a simplified cross-talk network termed as mini network is established. The widely accepted key markers are selected to form the skeleton of the mini network. The cross-talk among key markers is modeled by transit compartments model. Robustness and the integral variation of the mini network are proposed to be used as a good proxy for complex disease progression. In our model, three mini network integral disruption parameters *U, K*, and φ are introduced to evaluate integral variation of the network and mini network disruption probability is employed to measure the robustness of mini network.

## Materials and method

### Subject

Data used in the preparation of this article were obtained from the Alzheimer's Disease Neuroimaging Initiative (ADNI) database (adni.loni.usc.edu). The initial goal of ADNI was to recruit 800 subjects but ADNI has been followed by ADNI-GO and ADNI-2. To date these three protocols have recruited over 1500 adults, ages 55–90, to participate in the research, consisting of cognitively normal older individuals, people with early or late MCI, and people with early AD. For up-to-date information, see http://www.adni-info.org.

In this study, the longitudinal biomarker data (4 years, version: 2012-09-06) in ADNI-1 is used to develop the model. The data set includes three CSF markers: Tau protein, Aβ, and Phosphorylated tau protein (P-tau). Details of the CSF analysis and quality control measures have previously been published (Shaw et al., 2009). The patients are divided into three groups: AD, MCI, and normal control group. The CSF samples are collected at four time points: 12, 24, 36, and 48 months. Patients with missing data are excluded in our experiments. The demographic information of subjects used in this study at different time points is given in Table 1.

**Table 1:** **Demographic information of subjects**.

	**Normal**	**MCI**	**AD**
Number	135	155	18
Gender (male/female)	71/60	106/49	6/12
Age	79 ± 5	77 ± 6	74 ± 7
MMSE	29 ± 1	27 ± 2	24 ± 2

### Model development

**Figure 2:**
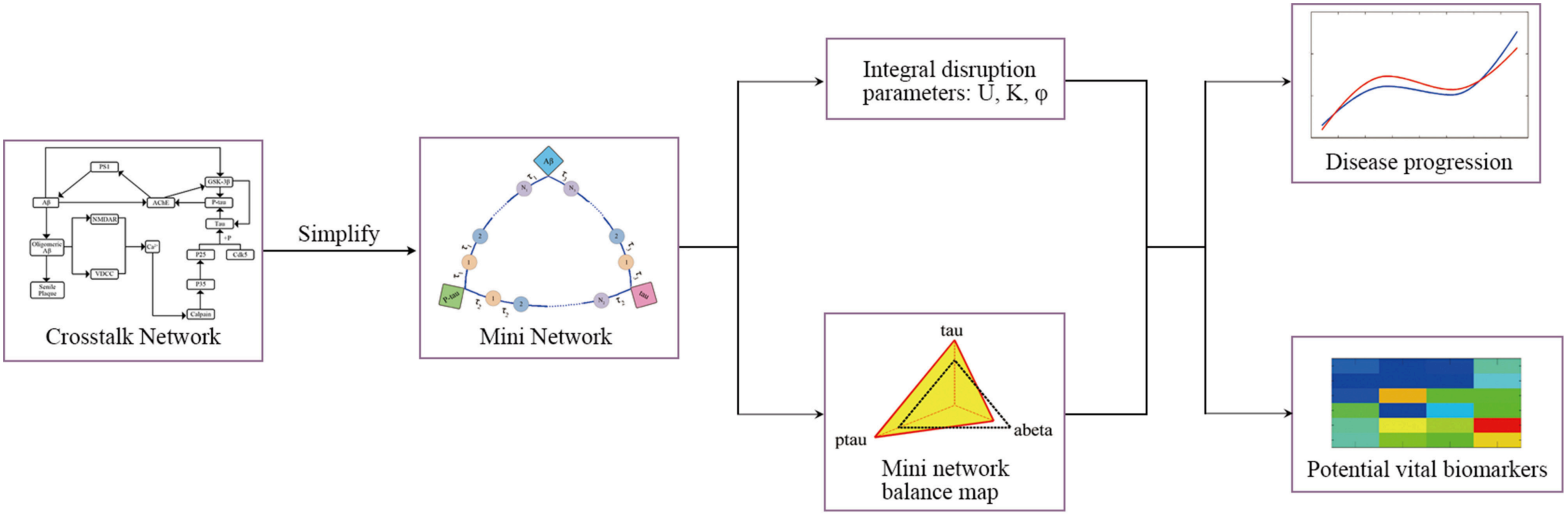
**The framework of model**.

In this study, the mini network is established based on the property of small-worldness. Then the robustness of the mini network is evaluated by Monte Carlo Simulation and disruption parameters. In addition, our model can identify the potential vital biomarkers in the disease progression. The framework of the model is shown in Figure 2.

#### Modeling mini network

**Figure 3:**
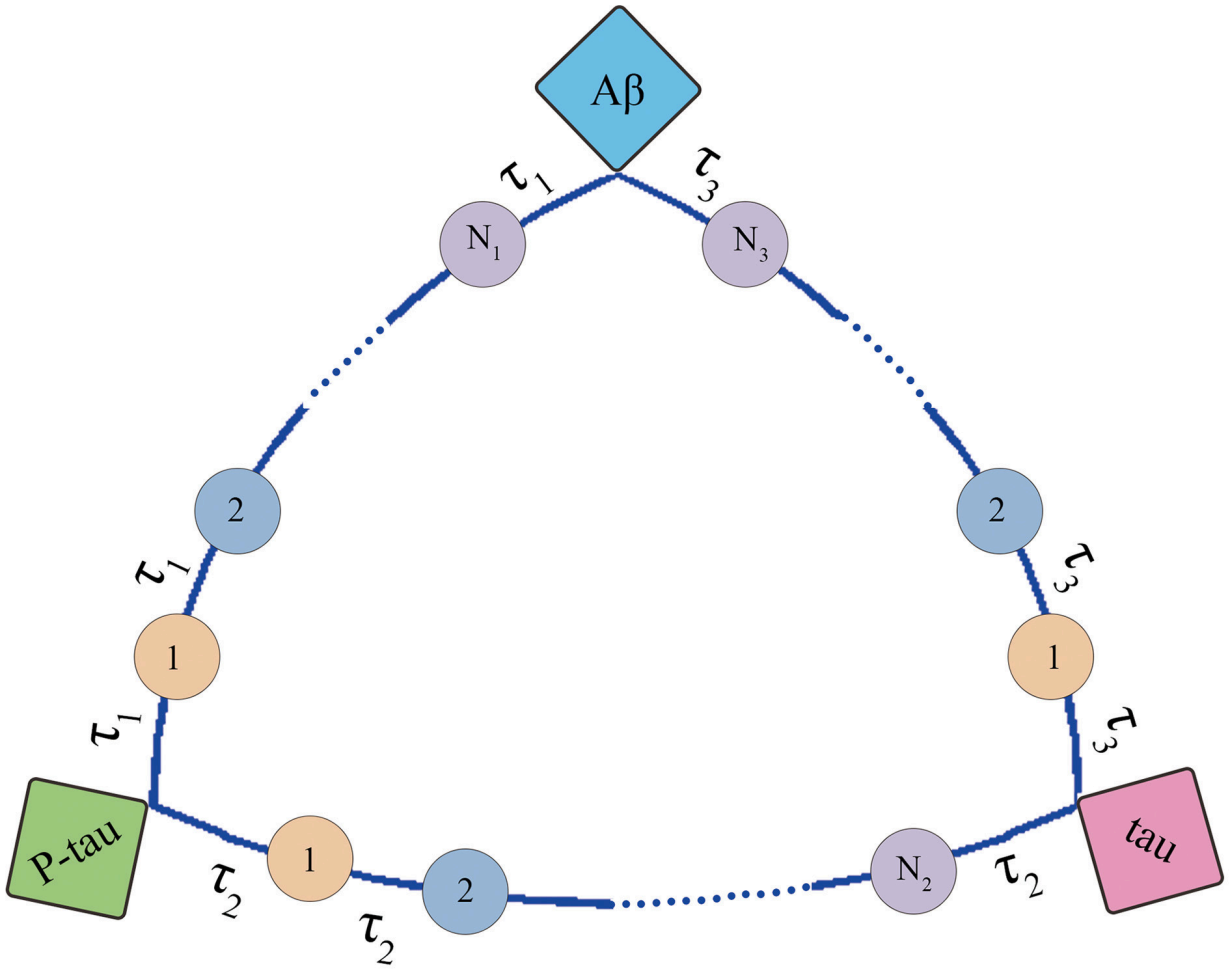
**Structure of mini network**. Transit compartments with a mean transit time constant τ are used to descript the indirect interactions between these markers.

The structure of mini network is shown in Figure 3. The mini network is established based on small-worldness property which means that the markers in the network can affect each other. The interaction among markers involves multiple middle links. For example, Aβ increases the activity of AChE. Then the AChE activates the GSK-3β inducing the hyperphosphorylation of tau. In contrast, P-tau can also affect Aβ by elevating AChE. In our model, these middle links between the biomarkers are represented by transit compartments. The mathematic model of mini network is given in Supplementary Material.

#### Estimation of mini network integral disruption parameters

In this study, three mini network integral disruption parameters *U, K*, and φ are used to evaluate the integral variation of the mini network.

(1) $$\begin{array}{l} K=\frac{\left|V_{a}\right|}{\left|V_{b}\right|} \end{array}$$

(2) $$\begin{array}{l} \text{φ}=\cos^{-1}\frac{V_{a}\cdot V_{b}}{\left|V_{a}\right|\left|V_{b}\right|} \end{array}$$

(3) $$\begin{array}{l} U=\sqrt{\left(V_{a}-V_{b}\right){\left(V_{a}-V_{b}\right)}^{T}} \end{array}$$

*V*_*a*_ is a vector including levels of all the markers in the mini network in pathogenic state. *V*_*b*_ is a vector including levels of all the markers in the mini network in normal state. |*V*_*a*_| and |*V*_*b*_| are their modular. The symbol “*T*” in Equation (3) represents vector transposition. Moreover, a simulation experiment is performed for investigating the physiological significance of the disruption parameters (shown in Investigation of Disruption Parameters Physiological Significance).

#### Estimation of mini network disruption probability

In this study Monte Carlo Simulation is used to estimate probability of mini network disruption. At the beginning, generate random perturbations for all the biomarkers in the mini network and assess the mini network disruption by *U, K*, and φ after random perturbation. Finally, calculate probability of network disruption (Equation 4) and its relative error (Equation 5), and repeat the above steps until the relative error is less than 5%.

(4) $$\begin{array}{rc} & p_{f}=\frac{d}{D} \end{array}$$

(5) $$\begin{array}{r} \frac{\varepsilon }{p}=t_{\alpha ∕2}\sqrt{\frac{p_{f}\left(1-p_{f}\right)}{p_{f}n}} \end{array}$$

*t*_α∕2_ is unilateral threshold of t distribution. *p*_*f*_ is the probability of mini network disruption. *n* is the predefined iterative number. *d* is number of network disruption. *D* is the current iterative number.

#### Recognition of potential vital biomarkers

We define the marker with the greatest contribution to the mini network disruption as the potential vital biomarker during the disease progression, and its contribution can be measured by the probability of mini network disruption calculated when only a single marker or a group of markers with interaction is disturbed. For the recognition of potential vital biomarkers, the first step of Monte Carlo simulation needs a minor modification. When evaluating the contribution of the ith marker, it needs to be disturbed and the other markers remain invariant. The joint contribution of multi-marker can be estimated in the same way: disturb the group of the markers to be evaluated and keep the other markers constant.

#### Model performance evaluation

In this study, we evaluate model performance in two ways. First, check if the mini network disorder probability can be used as a proxy for the disease progression by performing regression analysis of mini network and MMSE.

Second, check if the model can improve the accuracy of AD diagnosis by comparing classification accuracy of AD vs. normal and MCI vs. normal. Two Support vector machines (SVM) are trained for measure the classification performance: SVM based on mini network disruption parameters (*U, K*, φ) and mini network disorder probability and SVM based on CSF markers (tau, P-tau, and Aβ). The classification performance is evaluated by 10-fold cross-validation.

#### Investigation of disruption parameters physiological significance

To illustrate significance of *U, K*, and φ, we perform a simulation experiment. The change of biomarkers is simulated in three cases. The three situations are shown as follows:

- Single marker changing simulation: Only one marker changes as the gradient.
- Multi-marker changing simulation 1: All the markers increase as the gradient. For instance, the gradient is 10%. At this situation, all three markers increase 10%.
- Multi-marker changing simulation 2: The first marker increases as the gradient and the other two markers decrease as the gradient. For instance, marker A increases 10% and markers B and C decrease 10%.

Finally, observe the change of the three dynamic parameters *U, K*, and φ.

For a further insight into the significance of mini network integral disruption parameters, an addition test is performed. Three SVMs are trained for evaluating the contribution of mini network integral disruption parameters in the classification: SVM based on *K* and φ for assessing the contribution of parameter *U*, SVM based on *K* and *U* for assessing the contribution of parameter φ, SVM based on φ and *U* for assessing the contribution of parameter *K*. Then observe the performance of these three SVMs.

## Result

### Model performance

**Figure 4:**
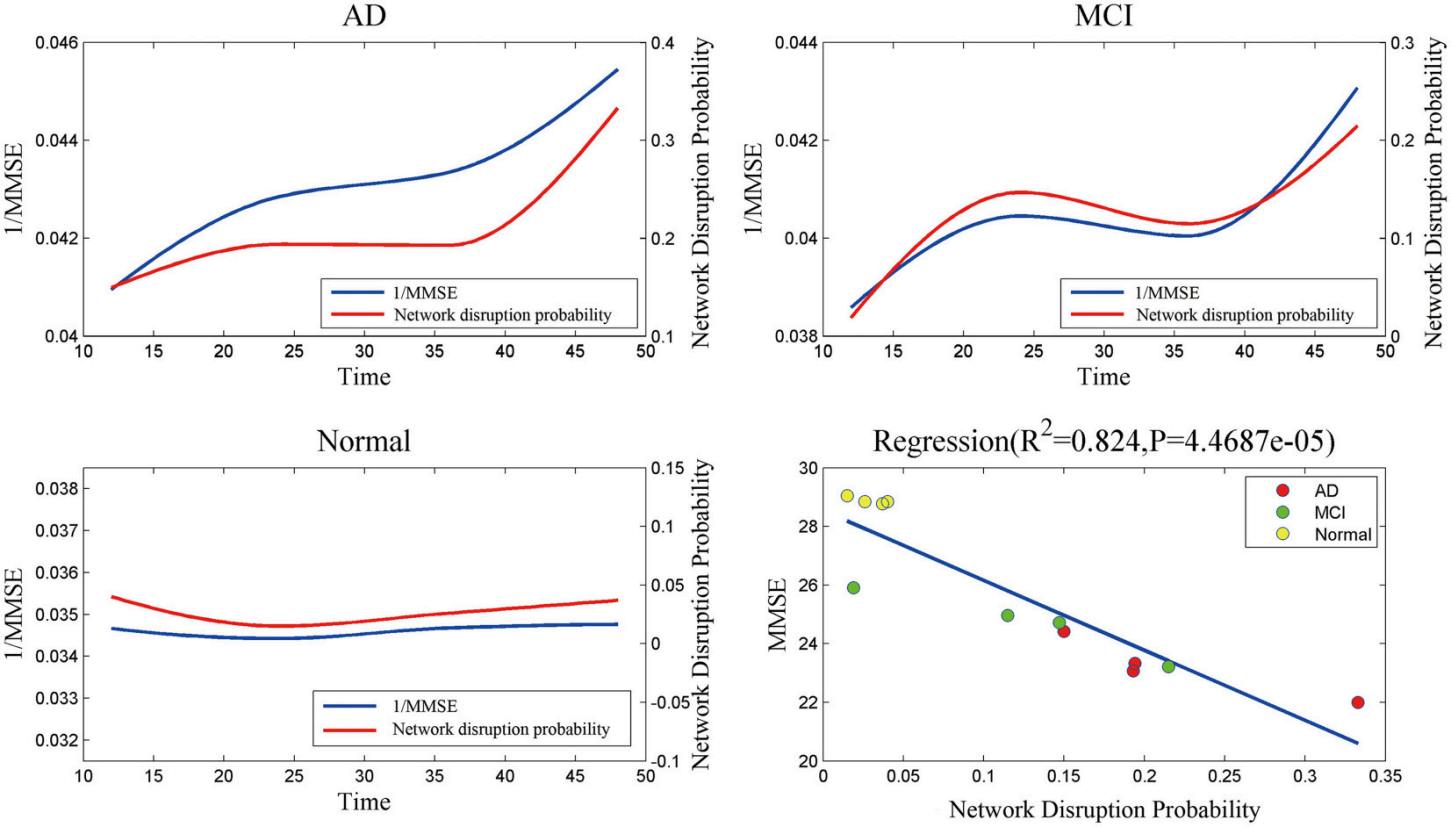
**The relationship and correlation analysis between estimated mini network disruption probability and MMSE**. As plot AD, MCI, and normal shown, mini network disruption probability coincides with 1/MMSE. In plot regression, least squares regression is used to analyze the correlation between mini network disruption probability and MMSE.

**Figure 5:**
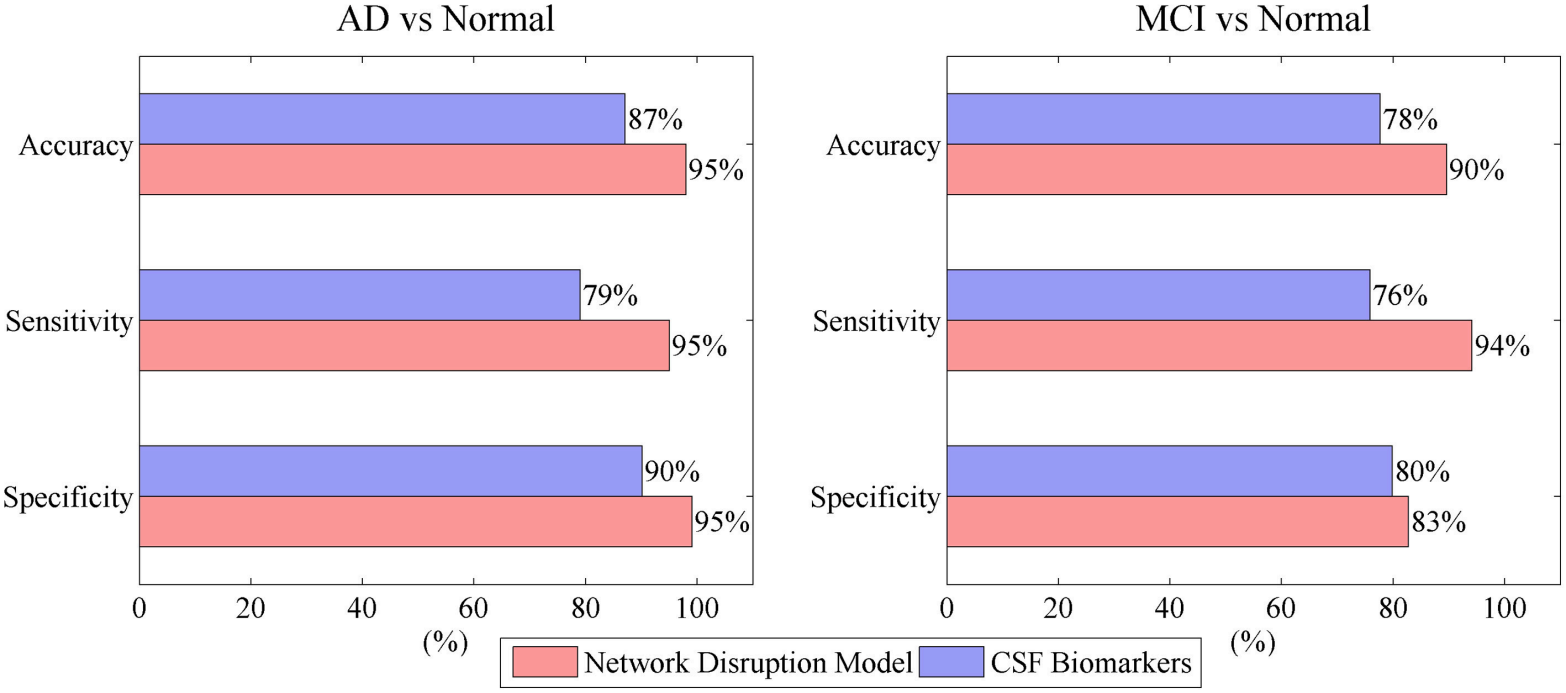
**Model performance in classification of AD vs. normal and MCI vs. normal**.

Figure 4 indicates that mini network disorder probability has good linear relationship with MMSE suggesting that mini network disorder may be a good proxy for disease progression. Figure 5 shows that our model may improve the classification accuracy and specificity of AD vs. normal and MCI vs. normal. Our model may enhance the classification sensitivity of MCI vs. normal.

### Evaluation of mini network balance

**Figure 6:**
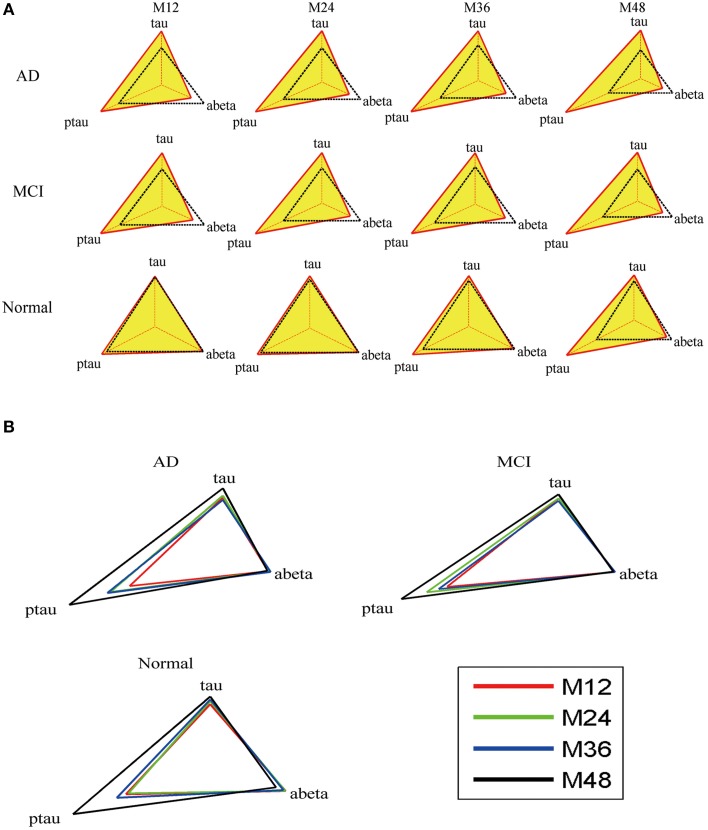
**Mini network balance map of AD based on CSF biomarkers panel. (A)** Mini network balance maps for the three groups at different time points. **(B)** Longitudinal mini network balance maps for the three groups. In normal state, the shape of mini network balance map is equilateral triangle. With the shape deformation, the mini network imbalance gets serious.

Mini network balance map (see Figure 6) is used to describe mini network imbalance visually. The closer to equilateral triangle the shape is, the less serious the mini network imbalance is. The results (Figure 6B) show that mini network imbalance deteriorates rapidly from 12 to 24 and 36 to 48 months while there is a plateau between the above two periods in both AD and MCI group, which coincide with the changes of MMSE.

The results of mini network disruption probability and MMSE are shown in the Figure 4. In AD group and MCI group, the mini network disruption probability increases rapidly between 12 and 24 months and there is a platform period from 24 to 36 months, then the disease progression turn into a rapid deterioration period until 48 months. In the normal control group, the mini network disruption probability keeps fluctuating during this study.

### Significance of mini network disruption parameters

The mini network disruption parameters *U, K*, and φ can be used to evaluate mini network imbalance integrally. According to Equations (1–3), with the mini network variation gets smaller, the *K*-value gets closer to 1 whereas the values of *U* and φ get closer to 0.

The simulation experiment result is presented in the Table 2. The results of single marker changing simulation indicate that the parameters *U* and φ are related to the variation of single marker. The results of multi-marker changing simulation 1 suggest that parameter *K* is related to the consistency multi-marker changing and *U* is sensitive to the great consistency variation of multi-marker. The results of multi-marker changing simulation 2 indicate that parameters *U* and φ are related to the multi-marker inconsistency variation. We summarize physiological significance of *U, K*, and φ with the simulation experiment evidence. *U* is responses to both consistency variation and inconsistency variation comprehensively. *K* responds to multi-marker consistency variation. φ is response to the multi-marker inconsistency variation.

**Table 2:** **The mini network integral disruption parameters changes in simulation experiment**.

	**Gradient (%)**	**K (%)**	**φ (%)**	**U (%)**
Situation: single marker simulation	10	3.78	12.88	11.09
	20	7.77	25.07	23.45
	50	20.77	57.21	65.17
Situation: multi-markers simulation 1	10	10.00	0.00	0.27
	20	20.00	0.00	6.82
	50	50.00	0.00	57.85
Situation: multi-markers simulation 2	10	2.19	27.68	25.30
	20	3.38	57.21	51.38
	50	0.82	147.97	131.69

**Figure 7:**
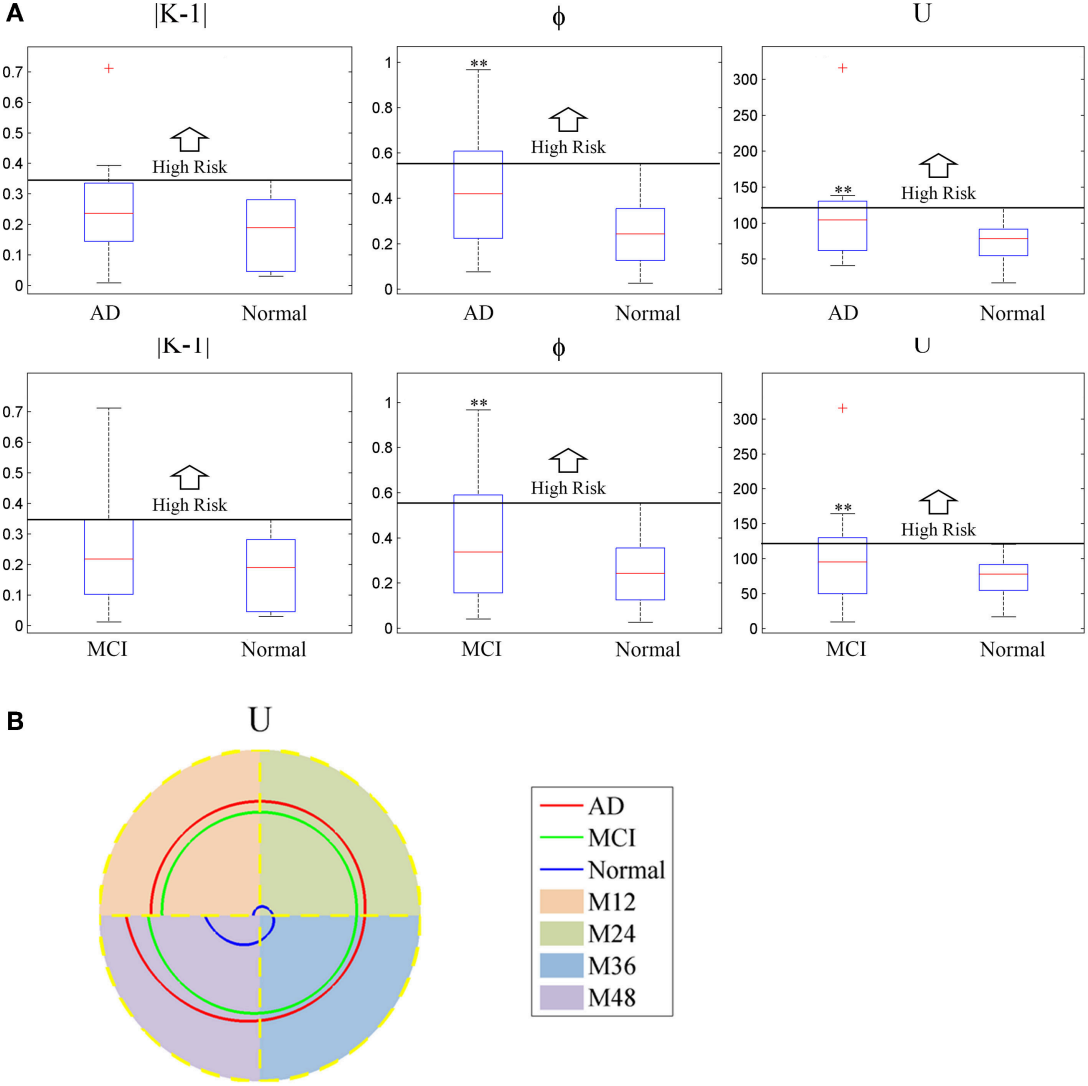
**(A)** Box plot of mini network integral disruption parameters. “+” represents data points beyond the whiskers. **(B)** The trajectory figure of the mini network integral disruption parameter *U*. The different colors of the area under the curve indicate different time period. With the curves of *U* farther to the center, mini network imbalance gets worse. ^**^P < 0.01 vs. normal.

The boxplot of mini network disruption parameters (Figure 7A) shows that these three parameters in both AD and MCI groups are significantly greater than those in normal group (*P* < 0.01, based on One-way ANOVA, Table 3) which suggests that if mini network disruption parameters *U, K*, and φ are higher than the upper whisker of normal group, the patient may have high disease risk. Moreover, the trajectory figure (Figure 7B) shows that the variation of parameter *U* is similar to the disease progression.

**Table 3:** **Estimation of disruption parameters *U, K*, and φ**.

	**U**	**K**	**φ**
AD	118.84 ± 87.12^**^	0.9684 ± 87	0.4484 ± 87^**^
MCI	103.80 ± 72.64^**^	0.9480 ± 72	0.3980 ± 72^**^
Normal	72.43 ± 30.35	0.923 ± 30	0.263 ± 30

*The data are presented as mean ± SD*.

** *P < 0.01 vs. Normal*.

**Figure 8:**
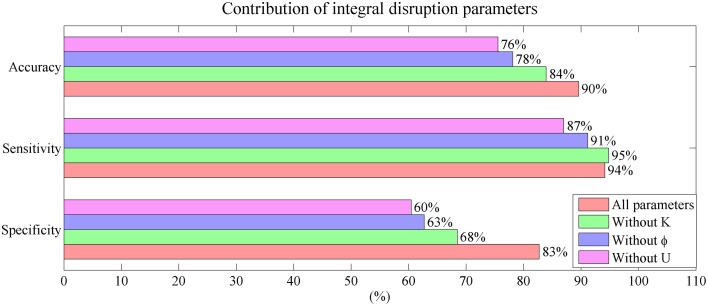
**Mini network integral disruption parameters' contribution to the performance in classification**.

The contribution of the mini network integral disruption parameters are shown in the Figure 8. The SVM based on all three parameters has the best classification performance compared with the other SVMs. The SVM without parameter *U* has the poorest performance which is same as the SVM based on CSF markers.

### Contribution of biomarkers to mini network disruption

**Figure 9:**
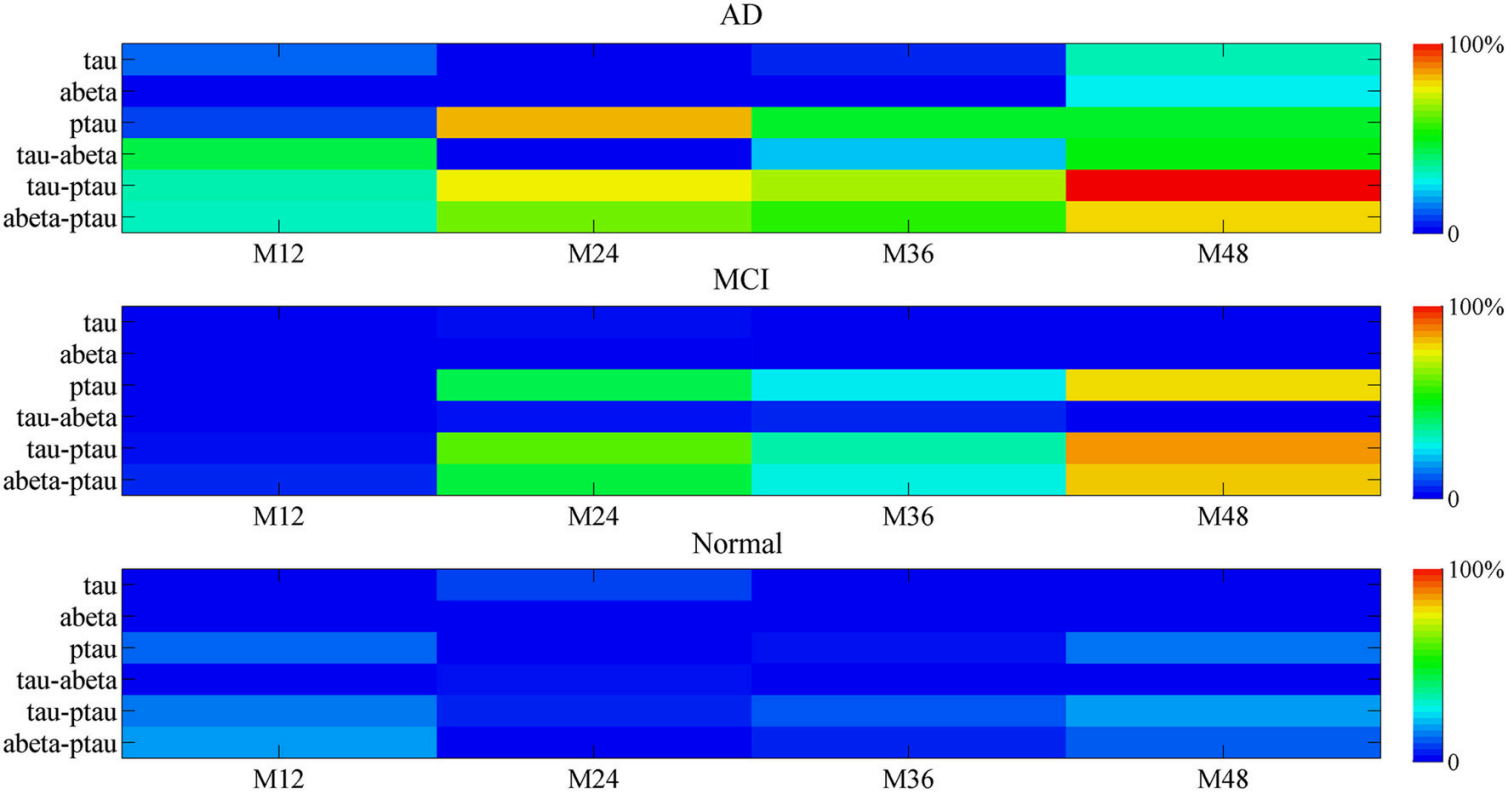
**The percentage contribution to mini network disruption of the markers**. Color-coded scale is used to present the percentage contribution to mini network disruption of the markers in this plot. The joint contribution of tau and P-tau as well as P-tau and Aβ both are potential vital factors of disease.

The biomarker contribution to the mini network disruption is given in the Figure 9. For the single marker, P-tau is the major contributor to the mini network in the disease progression in both AD and MCI. However, effects of other two markers Aβ and tau on aggravating mini network disruption cannot be neglected at the end stage (at 48 month). The joint contribution of P-tau and Aβ may play a more important role in the deterioration of the disease.

## Discussion

In this study, we propose a disease progression model termed as mini network balance model. The mini network balance model evaluates the disease progression in three ways. Firstly, the mini network balance map describes mini network imbalance visually. Secondly, the robustness of mini network is measured by mini network disruption probability which is a proxy for the disease progression. Thirdly, the integral variation of mini network is evaluated by the mini network disruption parameters *U, K*, and φ. The variation of mini network is usually complex. These three parameters decompose the complicated variation into simple variation. Firstly, parameter *K* is response to the consistency variation of biomarkers in mini network. Secondly, parameter φ represents the inconsistency variation of biomarkers in mini network. Parameter *U* is response to the total variation. With value of mini network integral parameters greater, the disease risk gets higher. The clinical relevance of mini network integral disruption parameters is that they can help enhance the accuracy and specificity of AD and MCI diagnosis. Especially, parameter *U* has the greatest contribution to the accuracy and specificity of the classification.

Compared to several previous researches based on CSF markers list in Table 4, the mini network balance model achieves relatively high performance on the classification of AD vs. normal and MCI vs. normal. Furthermore, the comparison to researches based on imaging markers shows that CSF markers and imaging markers may play similar roles in classification of AD vs. normal. Because, AD may cause atrophy in multiple region in brain and CSF markers reflect these change (Reiman and Jagust, 2012; Rosenmann, 2012). However, these two different types may play different roles in classification of MCI vs. normal. On one hand, imaging methods are often recommended to help rule out potentially reversible brain abnormalities like tumors or subdural hematomas in patients with MCI (Reiman and Jagust, 2012). On the other hand, CSF markers have better accuracy and specificity in classification of MCI vs. normal and they can be employed to identify the prodromal AD (Blennow and Zetterberg, 2015).

**Table 4:** **Comparison to previous researches**.

**Research**	**Biomarkers**	**Normal vs. AD**			**Normal vs. MCI**		
		**ACC (%)**	**SEN (%)**	**SPE (%)**	**ACC (%)**	**SEN (%)**	**SPE (%)**
SVM based on network disruption model (this study)	CSF	95	95	95	90	95	84
SVM (Apostolova et al., 2014)	CSF	82	–	–	74	–	–
Multi-modal multi-task (M3T) learning (Zhang et al., 2012)	CSF	93	–	–	83	–	–
Logistic Regression (Teipel et al., 2007)	CSF	81	78	83	–	–	–
Large-scale regularized logistic regression (Casanova et al., 2013)	CSF	75	71	78	64	50	74
Multi-modal multi-task (M3T) learning (Zhang et al., 2012)	MRI	93	–	–	83	–	–
SVM (Salvatore et al., 2015)	MRI	76	–	–	72	–	–
Sparse representation (Xu et al., 2015)	MRI	95	96	90	75	66	82
Image-level hierarchical classifier learning (Suk et al., 2014)	MRI	92	92	95	84	99	54
Multi-modal multi-task (M3T) learning (Zhang et al., 2012)	PET	93	–	–	83	–	–
Sparse representation (Xu et al., 2015)	PET	91	89	93	72	65	79
Image-level hierarchical classifier learning (Suk et al., 2014)	PET	92	88	96	84	99	57

*ACC, accuracy; SEN, sensitivity; SPE, specificity*.

The amyloid cascade hypothesis has been widely accepted as AD etiology. However, our model shows that Aβ accumulation may not be the sole factor in AD etiology. Joint contribution of P-tau and Aβ may be another potential major contributor to the AD progression. Consistent with Prior studies, Aβ toxicity is P-tau dependent (Kayed, 2010; Desikan et al., 2012). In other word, Aβ in the absence of P-tau is not necessarily associated with loss of cognitive function. Previous researches have proposed a possible mechanism for the interaction of P-tau and Aβ. On one hand, P-tau can increase the activity of AChE which can elevate the level of PS-1 and then accelerate the Aβ deposition (García-Ayllón et al., 2011; Silveyra et al., 2012). On the other hand, Aβ can activate tau hyperphosphorylation pathways (García-Ayllón et al., 2011). The crosstalk between Aβ and P-tau forms a vicious cycle in which they elevate each other and trigger cognitive decline.

Furthermore, more and more evidences in clinical trials suggest the multifactorial nature of AD. For example, Anti-bodies bapineuzumab and solanezumab targeted at Aβ have failed to meet their primary endpoints in the high-profile phase 3 clinical trials (Castello et al., 2014). Doody et al. attribute the failure to administering the therapy at late stages and propose that it should be instituted in early stages (Doody et al., 2014). However, the proposed model suggests that even at early stage the therapies targeted at Aβ only may have limited effect on AD. The presented results suggest that P-tau may also play a vital role in AD progression. Several prior studies have proved that immunotherapies targeted at P-tau can reduce cognitive impairment in animal model (Boutajangout et al., 2010; Kayed, 2010; Lim et al., 2013). In addition, our results suggested that single marker might play a limited role in disease progression and joint contribution of P-tau and Aβ might be the potential vital factors in the disease progression. Therefore, the single-target immunotherapies may have limited effects on AD. According to our results, combination therapy of reducing both P-tau and Aβ may be an effective strategy for AD treatment.

## Conclusion

This paper provides a novel method for modeling the complex disease progression. The mini network balance model has good performance on evaluating the AD progression which is beneficial on AD early diagnosis and facilitating therapeutic strategies.

## Author contributions

HL, CW, HH, and XL developed the model. HL and CW do the computational work. HL wrote the manuscript.

### Conflict of interest statement

The authors declare that the research was conducted in the absence of any commercial or financial relationships that could be construed as a potential conflict of interest.

## Footnotes

^1^Data used in preparation of this article were obtained from the Alzheimer's Disease Neuroimaging Initiative (ADNI) database (adni.loni.usc.edu). As such the investigators within the ADNI contributed to the design implementation of ADNI and/or provided data but did not participate in analysis or writing of this report. A complete listing of ADNI investigators can be found at: http://adni.loni.usc.edu/wp-content/uploads/how_to_apply/ADNI_Acknowledgement_List.pdf
