# Supplementary material for: Evaluating Alzheimer's Disease Progression by Modeling Crosstalk Network Disruption
Source: Front Neurosci. 2016 Jan 19;9:523. doi: 10.3389/fnins.2015.00523 (PMC4718081; doi:10.3389/fnins.2015.00523)
Supplement: Supplementary file 1 [file Presentation1.PDF]

Supplementary Material

Evaluating Alzheimer's disease progression by modeling crosstalk network disruption

Haochen Liu[1], Chunxiang Wei[1], Hua He[1,*], Xiaoquan Liu[1,*], for the Alzheimer's Disease Neuroimaging Initiative[**]

[1] *Center of Drug Metabolism and Pharmacokinetics, China Pharmaceutical University, Nanjing, 210009, China*

[*] *Corresponds author*

Tel.: + 86-25-83271260

E-Mail address: *lxq@cpu.edu.cn* (XQL), huahe827@163.com (HH).

** Data used in preparation of this article were obtained from the Alzheimer's Disease Neuroimaging Initiative (ADNI) database (adni.loni.usc.edu). As such, the investigators within the ADNI contributed to the design and implementation of ADNI and/or provided data but did not participate in analysis or writing of this report. A complete listing of ADNI investigators can be found at:

http://adni.loni.usc.edu/wp-content/uploads/how_to_apply/ADNI_Acknowledgement_List.pdf

The transit compartments are used to link different biomarkers. In the network, the variation of markers will impose disturbances then influence the other markers. We assume that perturbation can transit from one biomarker to another through the transit compartments and attenuate during spreading in the transit compartments. Therefore, the mini network is described as the following equations:

$$\begin{cases} \ln(\frac{1}{\Delta c_{tau}}) = w_{tau\leftarrow P-tau}\lambda_{tau\leftarrow P-tau}\Delta c_{P-tau} + w_{tau\leftarrow A\beta}\lambda_{tau\leftarrow A\beta}\Delta c_{A\beta} \\ \ln(\frac{1}{\Delta c_{P-tau}}) = w_{P-tau\leftarrow tau}\lambda_{P-tau\leftarrow tau}\Delta c_{tau} + w_{P-tau\leftarrow A\beta}\lambda_{P-tau\leftarrow A\beta}\Delta c_{A\beta} \\ \ln(\frac{1}{\Delta c_{A\beta}}) = w_{A\beta\leftarrow P-tau}\lambda_{A\beta\leftarrow P-tau}\Delta c_{P-tau} + w_{A\beta\leftarrow tau}\lambda_{A\beta\leftarrow tau}\Delta c_{tau} \end{cases} \quad (1)$$

Δc_{tau}, Δc_{P-tau}, and $\Delta c_{A\beta}$ are the percentage changing of tau, P-tau, and Aβ. $\lambda_{A\leftarrow B}$ indicates the loss of perturbation form marker B to marker A (e.g. The change of P-tau imposes perturbations which will transit to tau and $\lambda_{tau\leftarrow P-tau}$ represents the attenuation of perturbation from P-tau to tau). $w_{A\leftarrow B}$ is the interaction factor which represents the effect of marker B on marker A (e.g. the perturbation from P-tau can affect tau. $w_{tau\leftarrow P-tau}$ indicates the effect of P-tau on tau). To estimate parameters $\lambda_{A\leftarrow B}$ and $w_{A\leftarrow B}$ two steps are included: firstly estimating λ by Markov chain Monte Carlo (MCMC), secondly, estimating $w_{A\leftarrow B}$ by fitting the generalized linear model (GLM).

The parameter $\lambda_{A\leftarrow B}$ is related to the amount of compartments $N_{A\leftarrow B}$ and the average transition time $\tau_{A\leftarrow B}$:

$$\lambda_{A\leftarrow B} = (\frac{1}{\tau_{A\leftarrow B}})^{N_{A\leftarrow B}} \quad (2)$$

Considering that the target biomarker is disturbed by only one maker, the target biomarker concentration percentage changing (Δc_i) follows the Poisson distribution. In this study we assume that perturbation comes from multiple markers in the mini network, Δc_i obeys a joint distribution:

$$f(\Delta c_i) \propto \prod_i \frac{\lambda_{A\leftarrow B}^{\Delta c_i}}{\Delta c_s!} \propto \frac{\lambda^{\Delta c_i}}{\Delta c_i!} e^{-\lambda_{A\leftarrow B}} \qquad (3)$$

and

$$\lambda = \sum \left(\frac{1}{\tau_{A\leftarrow B}}\right)^{N_{A\leftarrow B}} \qquad (4)$$

Δc_s is the percentage changing of the sth marker imposed disturbance to the target marker Δc_i. The subscripted i and s can be 1 to 3.

The procedure of MCMC is shown as follow:

1. Construct the prior distributions of parameters to be estimated.
2. Derive the posterior distributions from the prior distributions.
3. Generate Markov chains using sampling algorithm.
4. Get the estimate parameters from the Markov chains.

We assume that $N_{A\leftarrow B}$ follows the Poisson distribution:

$$p(N_{A\leftarrow B}) = \frac{\tau_{A\leftarrow B}^{N_{A\leftarrow B}}}{N_{A\leftarrow B}} e^{-\tau_{A\leftarrow B}} \qquad (5)$$

And $\tau_{A\leftarrow B}$ obeys a uniform prior:

$$p(\tau_{A\leftarrow B}) \propto 1 \qquad (6)$$

The joint posterior distribution can be generated from prior distributions:

$$\pi(\Theta\,|\,\Delta C_i) \propto \prod_j \frac{\left(\sum\left(\frac{1}{\tau_{A\leftarrow B}}\right)^{N_{A\leftarrow B}}\right)^{\Delta c_i^j}}{\Delta c_i^j} e^{-\sum\left(\frac{1}{\tau_{A\leftarrow B}}\right)^{N_{A\leftarrow B}}} \times \prod \frac{\tau_{A\leftarrow B}^{N_{A\leftarrow B}}}{N_{A\leftarrow B}} e^{-\tau_{A\leftarrow B}} \qquad (7)$$

where Θ is estimated parameter set. Δc_i^j is the ith biomarker concentration percentage changing of the jth subject. The posterior distribution of $\tau_{A\leftarrow B}$ and $N_{A\leftarrow B}$ is derived by Eq.7:

$$\pi(N_{A\leftarrow B}\,|\,\Theta(\tau),\Theta^*(N),\Delta C_i) \propto \frac{\left(\sum\left(\frac{1}{\tau_{A\leftarrow B}}\right)^{N_{A\leftarrow B}}\right)^{\sum_j \Delta c_i^j} \tau_{A\leftarrow B}^{N_{A\leftarrow B}}}{N_{A\leftarrow B}\,!} e^{-\left(\tau_{A\leftarrow B} + j\sum\left(\frac{1}{\tau_{A\leftarrow B}}\right)^{N_{A\leftarrow B}}\right)} \qquad (8)$$

$$\pi(\tau_{A\leftarrow B}\,|\,\Theta(N),\Theta^*(\tau),\Delta c_i) \propto \left(\sum\left(\frac{1}{\tau_{A\leftarrow B}}\right)^{N_{A\leftarrow B}}\right)^{\sum_j \Delta c_i^j} \tau_{A\leftarrow B}^{N_{A\leftarrow B}} e^{-\left(\tau_{A\leftarrow B} + j\sum\left(\frac{1}{\tau_{A\leftarrow B}}\right)^{N_{A\leftarrow B}}\right)} \qquad (9)$$

$\Theta(\tau)$ and $\Theta(N)$ are estimated parameter sets including all the estimated τ and *N*. $\Theta^*(N)$ and $\Theta^*(\tau)$ are estimated parameter sets including all the estimated τ and *N* except $N_{A\leftarrow B}$ and $\tau_{A\leftarrow B}$. Parameter j is the simple size of subjects. Then Metropolis–Hastings (MH) algorithm is used to generate Markov chains with equilibrium distributions $\pi(N_n\,|\,\Theta(\tau),\Theta^*(N),\Delta C_0)$ and $\pi(\tau_n\,|\,\Theta(N),\Theta^*(\tau),\Delta C_0)$.

Supplemental figures:

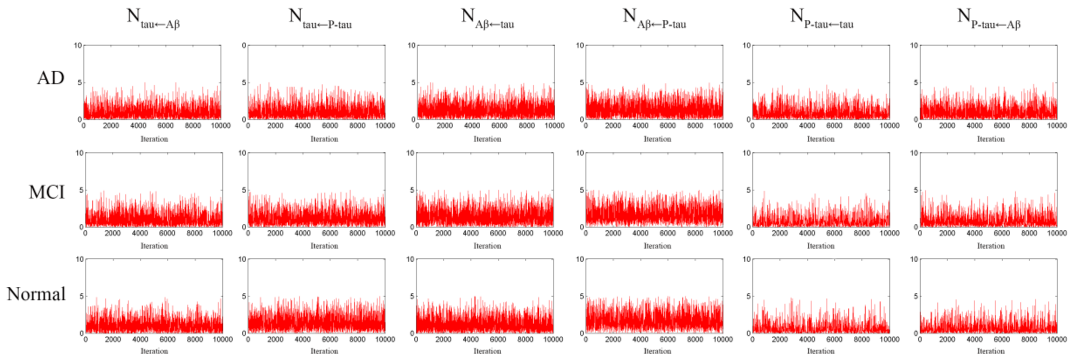

Fig. 1 Parameters $N_{tau\leftarrow A\beta}$, $N_{tau\leftarrow P-tau}$, $N_{A\beta\leftarrow tau}$, $N_{A\beta\leftarrow P-tau}$, $N_{P-tau\leftarrow tau}$, $N_{P-tau\leftarrow A\beta}$ in groups AD, MCI and normal at time point M12 versus iterations.

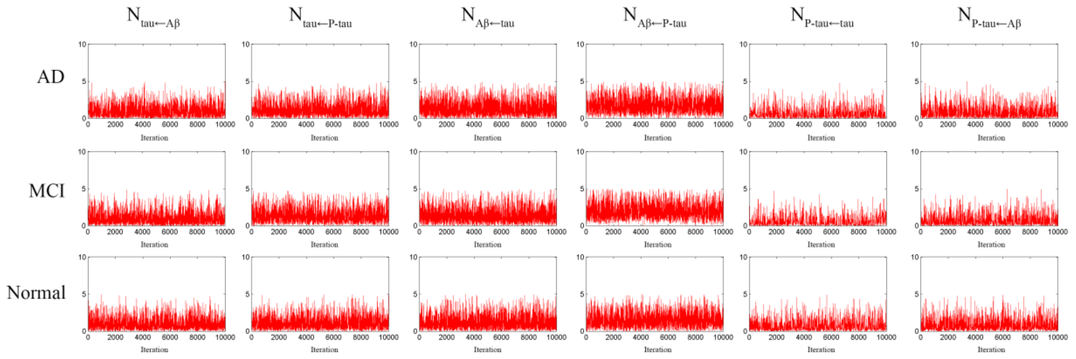

Fig. 2 Parameters $N_{tau \leftarrow A\beta}$, $N_{tau \leftarrow P-tau}$, $N_{A\beta \leftarrow tau}$, $N_{A\beta \leftarrow P-tau}$, $N_{P-tau \leftarrow tau}$, $N_{P-tau \leftarrow A\beta}$ in groups AD, MCI and normal at time point M24 versus iterations.

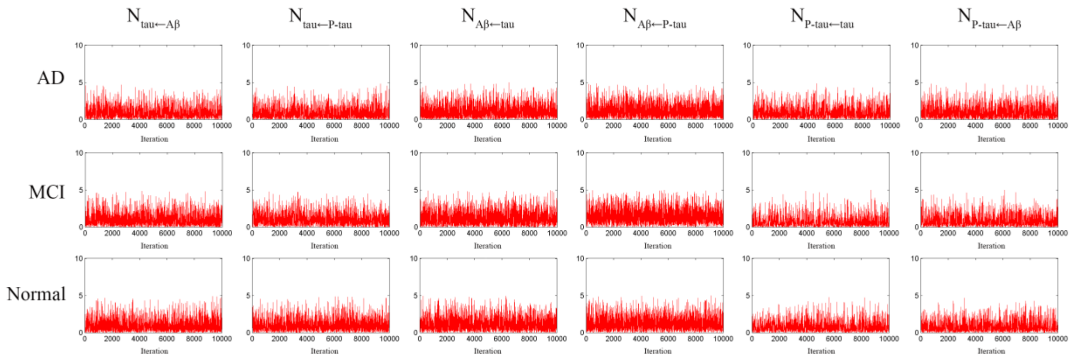

Fig. 3 Parameters $N_{tau \leftarrow A\beta}$, $N_{tau \leftarrow P-tau}$, $N_{A\beta \leftarrow tau}$, $N_{A\beta \leftarrow P-tau}$, $N_{P-tau \leftarrow tau}$, $N_{P-tau \leftarrow A\beta}$ in groups AD, MCI and normal at time point M36 versus iterations.

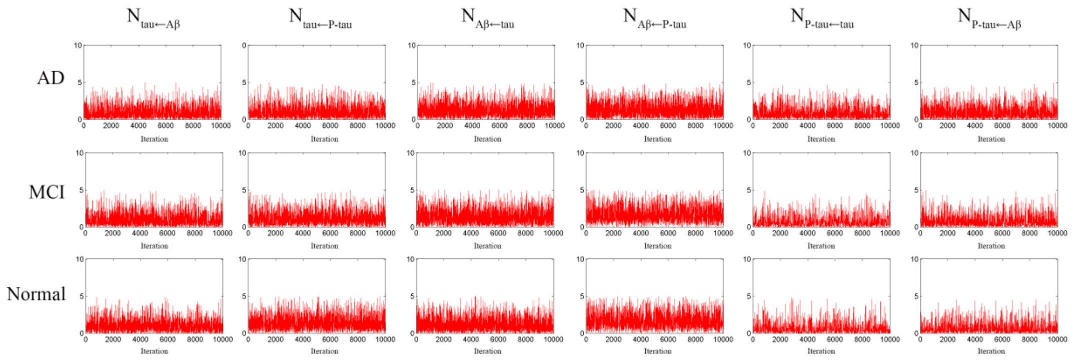

Fig. 4 Parameters $N_{tau \leftarrow A\beta}$, $N_{tau \leftarrow P-tau}$, $N_{A\beta \leftarrow tau}$, $N_{A\beta \leftarrow P-tau}$, $N_{P-tau \leftarrow tau}$, $N_{P-tau \leftarrow A\beta}$ in groups AD, MCI and normal at time point M48 versus iterations.

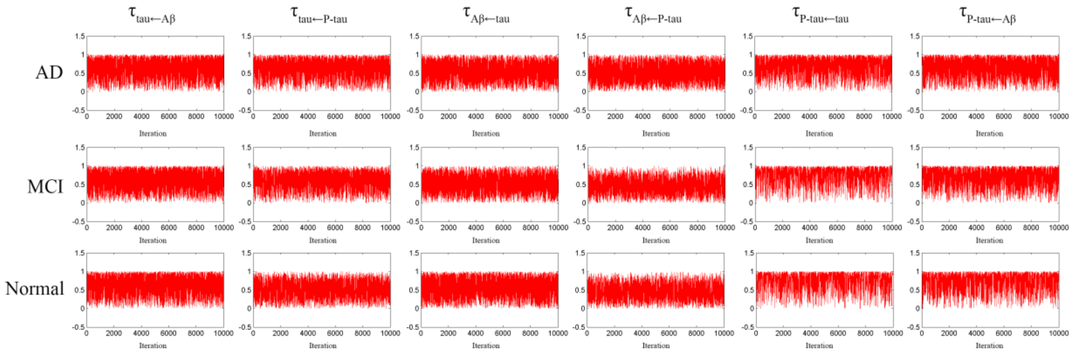

Fig. 5 Parameters $\tau_{tau\leftarrow A\beta}$, $\tau_{tau\leftarrow P-tau}$, $\tau_{A\beta\leftarrow tau}$, $\tau_{A\beta\leftarrow P-tau}$, $\tau_{P-tau\leftarrow tau}$, $\tau_{P-tau\leftarrow A\beta}$ in groups AD, MCI and normal at time point M12 versus iterations.

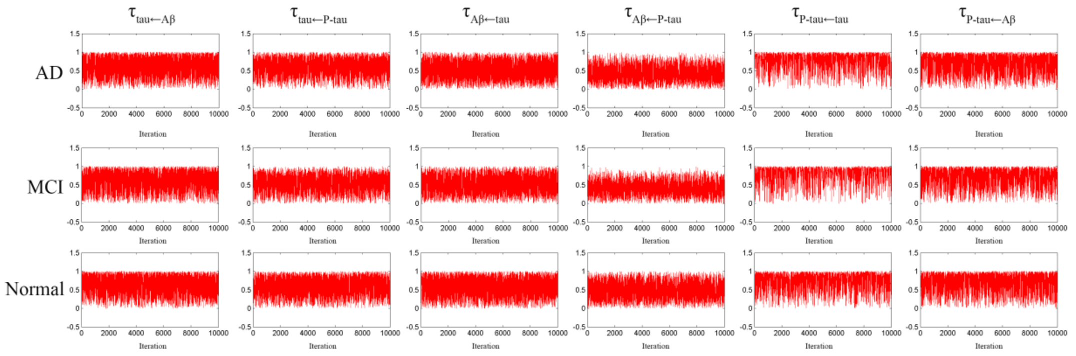

Fig. 6 Parameters $\tau_{tau\leftarrow A\beta}$, $\tau_{tau\leftarrow P-tau}$, $\tau_{A\beta\leftarrow tau}$, $\tau_{A\beta\leftarrow P-tau}$, $\tau_{P-tau\leftarrow tau}$, $\tau_{P-tau\leftarrow A\beta}$ in groups AD, MCI and normal at time point M24 versus iterations.

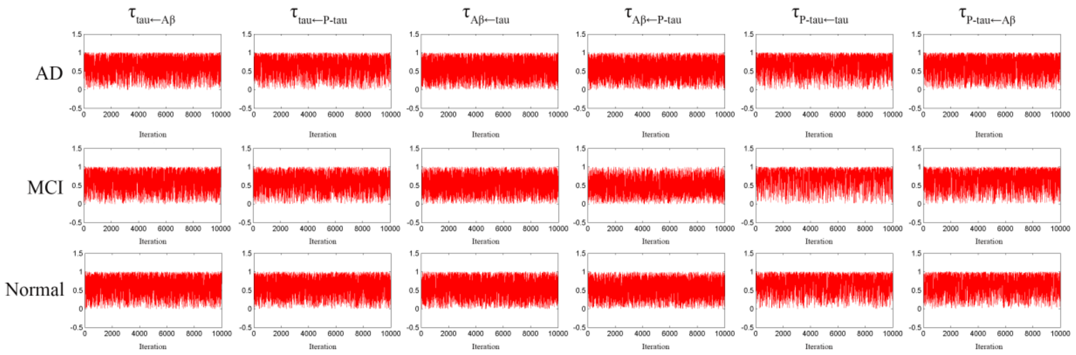

Fig. 7 Parameters $\tau_{tau\leftarrow A\beta}$, $\tau_{tau\leftarrow P-tau}$, $\tau_{A\beta\leftarrow tau}$, $\tau_{A\beta\leftarrow P-tau}$, $\tau_{P-tau\leftarrow tau}$, $\tau_{P-tau\leftarrow A\beta}$ in groups AD, MCI and normal at time point M36 versus iterations.

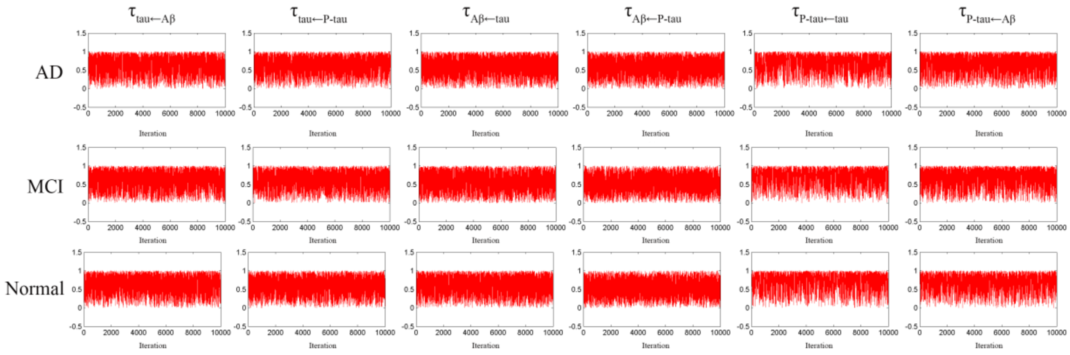

Fig. 8 Parameters $\tau_{tau \leftarrow A\beta}$, $\tau_{tau \leftarrow P-tau}$, $\tau_{A\beta \leftarrow tau}$, $\tau_{A\beta \leftarrow P-tau}$, $\tau_{P-tau \leftarrow tau}$, $\tau_{P-tau \leftarrow A\beta}$ in groups AD, MCI and normal at time point M48 versus iterations.
